# Peyer’s Patch B Cells Sample Transglutaminase-Gluten Complexes and Drive Celiac Disease Autoimmunity

**DOI:** 10.1053/j.gastro.2025.06.021

**Published:** 2025-07-01

**Authors:** M. Fleur du Pré, Liv Kleppa, Alisa E. Dewan, Maureen T. Meling, Lene S. Høydahl, Harrison A. Besser, Chaitan Khosla, Ludvig M. Sollid

**Affiliations:** 1 Norwegian Coeliac Disease Research Center, Institute of Clinical Medicine, University of Oslo, Oslo, Norway; 2 Department of Immunology, Oslo University Hospital - Rikshospitalet, Oslo, Norway; 3 Department of Chemistry, Stanford University, Stanford, California; 4 Stanford Medical Scientist Training Program, Stanford University School of Medicine, Stanford, California; 5 Department of Chemical Engineering and Sarafan ChEM-H, Stanford University, Stanford, California

**Keywords:** Celiac Disease, Transglutaminase 2, B Cells, Peyer’s Patch, Mouse Model

## Abstract

**BACKGROUND & AIMS:**

The production of autoantibodies against the enzyme transglutaminase 2 (TG2) in celiac disease likely results from TG2-specific B cells receiving help from gluten-specific CD4^+^ T cells in gut-associated lymphoid tissues (GALT) via the formation of transient enzyme-substrate complexes formed between TG2 and gluten. Where in the body enzymatically active TG2 encounters gluten peptides remains unknown.

**METHODS:**

A model to study the celiac disease-relevant T-cell-B-cell interactions in GALT has been developed. Mice expressing HLA-DQ2.5 received TG2-specific B cells and gluten-specific T cells by adoptive transfer and were subsequently orally immunized with a model antigen containing the the B-cell and T-cell epitopes.

**RESULTS:**

Orally immunized HLA-DQ2.5 knockin mice developed TG2-specific gut immunoglobulin (Ig)A and serum IgG responses. Activated TG2-specific B cells were present in Peyer’s patches and in gut-draining mesenteric lymph nodes, resembling what is seen in human celiac disease. We demonstrate that TG2-specific B cells in Peyer’s patches sample TG2 when the protein is perfused into the gut lumen.

**CONCLUSION:**

The model supports a mechanism in which TG2-gluten complexes formed in the gut lumen are taken up by TG2-specific B cells in GALT. We propose that this pathway plays an important role in driving the anti-TG2 IgA autoantibody response in patients with celiac disease. The model provides a platform to explore novel approaches for celiac disease therapies.

## Graphical Abstract

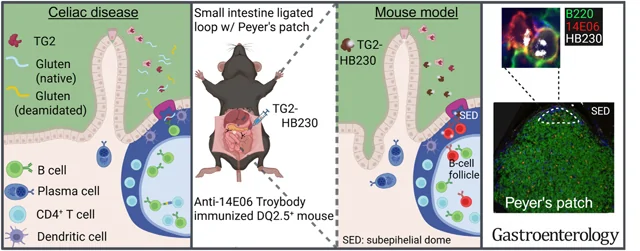

Celiac disease is a gluten-dependent enteropathy tightly associated with the formation of highly disease-specific autoantibodies. The pathogenesis is driven by CD4^+^ T cells that react with deamidated gluten peptides (DGPs) presented on the disease-associated human leukocyte antigen (HLA) molecules HLA-DQ2.5, HLA-DQ2.2, and HLA-DQ8.^1^ Patients with celiac disease have immunoglobulin (Ig)A and IgG autoantibodies in serum specific for the self enzyme transglutaminase 2 (TG2) as well as antibodies to DGP.^2^ In the small intestinal mucosae of patients with active celiac disease, there is a marked accumulation of IgA-producing plasma cells—of which 10% to 20% are TG2-specific compared with about 1% that are specific for DGP.^3–5^

Although both circulating anti-DGP and anti-TG2 antibodies are excellent diagnostic markers for celiac disease, secreted immunoglobulins are likely not involved in the development of the celiac enteropathy.^6^ Rather, B cells expressing the membrane-bound form of immunoglobulins, B-cell receptors (BCRs), are instrumental as antigen-presenting cells for gluten presentation to CD4^+^ T cells. The production of anti-TG2 antibodies is strictly dependent on dietary gluten exposure and these antibodies disappear within months when patients commence a gluten-free diet,^7^ which is the only available treatment for the disease. This gluten dependence strongly suggests the involvement of gluten-specific CD4^+^ T cells in the generation of anti-TG2 autoantibodies. TG2-specific B cells can capture enzyme-substrate intermediate complexes of TG2 and gluten, formed during the TG2-catalyzed deamidation reaction, via their B-cell receptors, thereby allowing for the uptake and processing of deamidated gluten antigens for presentation to gluten-specific CD4^+^ T cells.^8^ The interactions between gluten-specific CD4^+^ T cells and TG2-specific B cells not only result in the generation of TG2-specific autoantibodies and plasma cells, they also lead to a massive amplification of the inflammatory gluten-specific CD4^+^ T-cell response. Given the central role of the crosstalk between TG2-specific B cells and gluten-specific T cells in driving celiac pathology, disrupting this pathogenic crosstalk emerges as a possible therapeutic strategy.

An important unresolved question is where in the body the pathogenic interactions between TG2, gluten, and the T and B cells occur. Based on the observations that there are no signs of B-cell tolerance in transgenic mice expressing a celiac-patient derived anti-TG2 B-cell receptor,^9^ and that enterocytes contain abundant amounts of TG2,^10^ we proposed that pathogenic TG2-gluten enzyme-substrate complexes could be generated in the gut lumen and initiate an anti-TG2 antibody response in gut-associated lymphoid tissues (GALT). Within the GALT, Peyer’s patches (PPs) are the primary inductive sites for intestinal IgA responses.^11^ Luminal antigens are taken up through specialized microfold (M) cells of the follicle-associated epithelium into the sub-epithelial dome (SED) area of the PPs where they can be encountered by B cells that are found in pockets just below the M cells.^12^ These B cells can then present epitopes to T cells, resulting in the maturation and differentiation of the B cells that then migrate through the lymph via the thoracic duct into the blood, ultimately homing back to the gut to populate the small intestinal lamina propria as IgA plasma cells.^13^ Consistent with this notion, the specificity of gut IgA plasma cells reflects luminal antigens, and thus the high frequency of TG2-specific IgA plasma cells in the small intestinal mucosa of untreated celiac patients could indicate luminal TG2 as a priming factor for these plasma cells.

We have recently shown that TG2 derived from shed enterocytes can be detected in the gut lumen of mice.^14^ Luminal TG2 was catalytically active and able to generate DGPs, indicating that transient complexes of TG2 and gluten can be formed in the lumen of the small intestine.^14^ Whether luminal TG2 can initiate anti-TG2 autoimmunity in celiac disease remains to be determined. Here we addressed whether luminal TG2 antigens can be taken up by TG2-specific B cells in PPs. Thereto, we developed a model system to study TG2-specific B-cell responses in the gut using the anti-TG2 immunoglobulin knockin (KI) mouse.^9^ In an adoptive transfer model, TG2-specific B-cell responses are induced in a T-cell-dependent manner in PPs and gut-draining mesenteric lymph nodes following oral immunization. We found that TG2-specific B cells in the PP SED bound TG2 antigen injected into ligated loops of the small intestine, providing further support for a model in which luminal TG2 drives pathogenic T-cell-B-cell collaboration in GALT in celiac disease by catalyzing the formation of TG2-gluten complexes.

## Methods

### Mice

Anti-TG2 Ig (14E06) KI mice^9^ and T-cell receptor (TCR)-glia-*ω*2 transgenic mice^15^ crossed to HLA-DQ2.5 KI mice^16^ were bred at the Department of Comparative Medicine, Oslo University Hospital - Rikshospitalet, Oslo, Norway. HLA-DQ2.5 KI recipient mice in adoptive transfer experiments were crossed to CD45.1 mice (B6.SJL-*Ptprc*^a^ *Pepc*^b^/BoyCrl, Charles River Laboratories) and bred in-house. In some experiments, HLA-DQ2.5 KI recipients were also crossed to *Tgm2*^−/−^ mice.^17^ *Tgm2*^−/−^ 14E06 KI mice were used in all adoptive transfer experiments to avoid exposure of TG2-specific B cells to TG2 antigen released during tissue processing and cell isolation. All mice were on C57Bl/6 background and maintained on irradiated gluten-free mouse chow (D11112201i, Research Diets) in individually ventilated cages. Female and male mice 8 to 14 weeks old were used. Animal experiments were approved by the Norwegian Food Safety Authority (Mattilsynet) and conducted in accordance with Directive 2010/63/EU.

### Anti-14E06 Antibody and Anti-14E06-ω18 Troybody Production

Anti-14E06 Troybodies with the 18-mer ω-gliadin peptide sequence QPEQPFPQPEQPFPWQPQ containing the DQ2.5-glia-ω2 T-cell epitope (underlined) inserted into loop 6 of C_H_1 domain of mIgG1 or mIgG2b were generated. The binding of anti-14E06-ω18 L6CH1 Troybody and the anti-14E06 control antibody to human monoclonal antibody (hmAb) 14E06 was assessed by enzyme-linked immunosorbent assay (ELISA) and tested in in vitro T-cell-B-cell co-culture assay using transfected cell lines. See Supplementary Material and Methods for details.

### Adoptive Transfer and Immunization

Homozygous CD45.1 HLA-DQ2.5 recipient mice received 1 × 10^6^ TG2-specific B cells and 5 × 10^5^ gluten-specific CD4^+^ VRß^+^ T cells by intravenous injection in the lateral tail vein; for details see Supplementary Material and Methods. The next day, mice were immunized intragastrically with 250 to 400 *μ*g *α*-14E06-*ω*18 Troybody or anti-14E06 control antibody and/or 10 *μ*g cholera toxin (CT) (100B, List Biological Laboratories) in phosphate-buffered saline (PBS) with 3% (wt/vol) NaHCO_3_. Mice were fasted for 1 hour before oral immunization. Booster immunizations were given after 7 and 14 days. Analysis of anti-TG2 and anti-CT antibodies in serum and stool samples was conducted by ELISA. See Supplementary Material and Methods for details.

### Flow Cytometry

PPs were excised from the small intestine and carefully ground through a 70-*μ*m-diameter nylon mesh to prepare single-cell suspensions. Lymph nodes were cut with surgical scissors before single-cell suspensions were prepared in the same way. Cells were resuspended in PBS containing 2% fetal calf serum (vol/vol) and incubated with optimal amounts of the following antibodies: B220-APC-eFluor780 (1:200, 47–0452-82, eBioscience), B220-PerCP/Cy5.5 (1:200, 1032236, BioLegend), CD3-PerCP/Cy5.5 (1:50, 45–0031-82, eBioscience), CD4-APC-Cy7 (1:300, BioLegend, 100414), CD19-PE-Cy7 (1:200, 25–0193-82, eBioscience), CD38-eFluor450 (1:200, 48–038182, eBioscience), CD45.1-AlexaFluor647 (1:200, 110720, BioLegend) CD45.2-BV711 (1:200, 109847, BioLegend), CD95-PE-Cy7 (1:50, 557653, BD), CD185 (CXCR5)-APC (1:400, 145506, BioLegend), CD279 (PD-1)-PE-Cy7 (1:200, 25–9985-82, eBioscience), GL7-FITC (1:50, 144603, BioLegend), GL7-eFluor450 (1:100, 48–5902-82, eBioscience), IgD-BV605 (1:200, 405727, BioLegend), and IgM-APC-Cy7 (1:200, 406515, BioLegend). Unconjugated hIgG1 anti-14E06 (used at 0.55 *μ*g/mL) was produced as previously described^18^ and detected with phycoerythrin-conjugated anti-hIgG1 (1:200, AM08151RP-N, Acris Antibodies). Nonspecific antibody binding was blocked using an anti-FcγRIII/II antibody (anti-mouse CD16/32, 101302, BioLegend). Viability was assessed using 7-amino-actinomycin D (7-AAD, eBioscience). Cells were analyzed on Attune NxT Flow Cytometer (Thermo Fisher Scientific) and analysis was performed with FlowJo software (BD).

### Enzyme-Linked Immunosorbent Spot

Small intestinal lamina propria lymphocytes were isolated as described in detail in the Supplementary Material and Methods. MultiScreen HPS membrane plates (Millipore) were activated with 20 *μ*L 35% (vol/vol) ethanol for 1 minute, washed with sterile PBS, and coated overnight at 4C with 5 *μ*g/mL Neutravidin (Thermo Fisher Scientific) or anti-mouse IgA (556960, BD Pharmingen). For anti-CT enzyme-linked immunosorbent spot (ELISpot), plates were first incubated with 3 nmol/mL GM1-ganglioside (Merck) for 2 hours at room temperature, then washed with PBS and incubated with 0.2 *μ*g/mL CT overnight. After washing and blocking, Neutravidin-coated wells were incubated with 5 *μ*g/mL biotinylated human TG2 produced in Sf+ insect cells as previously described.^19^ Plates were washed and cells were added to plate in 3-fold serial dilutions in RPMI 1640 supplemented with 10% (vol/vol) fetal calf serum, 1% (vol/vol) penicillin/streptomycin, and 100 *μ*M β-mercaptoethanol and incubated for 3 hours at 37°C, 5% CO_2_. Plates were washed with PBS with Tween 20 (PBS-T) and incubated with anti-mouse IgA-AP (1:1000, 1040–04, Southern Biotech) overnight at 4°C. Plates were washed with PBS-T, PBS alone, and dH_2_O, and spots detected with NBT-5-bromo-4-chloro-3-indolyl phosphate substrate (Bio-Rad). Plates were rinsed thoroughly in H_2_O and dried overnight. Plates were scanned using a Cellular Technologies Limited (CTL) Immunospot Analyzer and spots quantified using the CTL Immunospot 5.1 software. Wells added medium only were used as negative controls.

### Immunohistochemistry

Approximately 0.5-cm pieces of small intestine containing a PP flushed with PBS were fixed in 4% paraformaldehyde and 10% sucrose for 1 hour on ice before transfer to 30% sucrose. Samples were kept at 4°C for 16 to 48 hours before embedding in TissueTek OCT compound and snap freezing in liquid nitrogen. Frozen tissue sections (7 *μ*m) were blocked with 2% IgG-free bovine serum albumin (Jackson ImmunoResearch) in PBS for 15 minutes. Antibodies used to stain tissue sections were anti-mouse B220-BV421 (1:100, 562922, BD), IgA-biotin (1:150, Southern Biotech, 1040–08), IgD-BV421 (1:50, 742286, BD), CD11c-BV421 (1:100, 562782, BD), CD254 (RANKL)-biotin (1:100, 13–5952-82, eBioscience), Ephrin-B1-biotin (1:50, BAF473, R&D Systems). Biotinylated antibodies were followed by Streptavidin-Alexa Fluor 488 (1:800, S32354, Invitrogen). hIgG1 anti-14E06 (used at 5 *μ*g/mL) was detected with donkey-anti-hIgG-Cy3 (1:400, 709–165-149, Jackson ImmunoResearch). Microscopy was performed using the Zeiss LSM 780 confocal microscope.

### Ligated Loop Assay

HLA-DQ2 KI mice were adoptively transferred with TG2-specific B cells and gluten-specific CD4^+^ T cells followed by 2 oral immunizations with mIgG1 *α*-14E06-*ω*18 Troy and CT. One week after the second immunization, the mice were anesthetized using isoflurane and an abdominal incision was made to expose the small intestine. PPs were identified and ligated loops containing 1 or 2 PPs were made and injected with 50 to 100 *μ*L TG2-HB230 at a concentration of 0.5 to 0.7 mg/mL in PBS supplemented with 5 mg/mL soybean trypsin inhibitor (Sigma-Aldrich) and 4 mM Pefabloc (Roche). To prepare TG2-HB230, recombinant mouse TG2 produced in *Escherichia coli* as previously described^9^ was incubated with the irreversible TG2 inhibitor HB230^20^ in Tris-buffered saline supplemented with 5 mM CaCl_2_ at a 1:3 molar ratio for 30 minutes on ice. After injection and ligation of the loop, the abdomen was closed with suture clips and the mice were kept under anesthesia for 2 hours. PPs were excised and prepared for microscopic analysis as described previously.

## Results

In mice, the gut antigen-specific IgA responses to orally administered antigens are efficiently induced when antigen is co-administered with CT as a mucosal adjuvant.^21^ Thus, to induce a TG2-specific IgA response in mice, we made use of our anti-TG2 immunoglobulin KI mouse (14E06 KI).^9^ We have previously shown that autoreactive TG2-specific B cells in these mice remain functionally silent, and that their activation and differentiation into autoantibody-producing plasma cells can be induced upon receiving T-cell help from gluten-specific CD4^+^ T cells in HLA-DQ2.5-expressing mice that are immunized systemically with a recombinant TG2-gluten fusion protein.^9^ As the previous study was done with splenic TG2-specific B cells but priming of TG2-specific B cells in patients with celiac disease presumably occurs in GALT, we here compared the frequency of TG2-specific B cells in PPs between *Tgm2*^+/+^ and *Tgm2*^−/−^ 14E06 KI mice. We found that also in PPs, most B cells express the autoreactive BCR and found no difference between *Tgm2*^+/+^ and *Tgm2*^−/−^ 14E06 KI mice (Supplementary Figure 1A). In agreement with our observations on splenic B cells,^9^ autoreactive TG2-specific B cells isolated from PPs of *Tgm2*^+/+^ 14E06 KI mice had somewhat lower expression of IgM on the cell surface when compared with TG2-specific B cells isolated from PPs of *Tgm2*^−/−^ 14E06 KI mice (Supplementary Figure 1B), which could very well be explained by exposure of the B cells to TG2 released from the cytosol during tissue processing. These data suggest that there is no tolerization of autoreactive TG2-specific B cells localized in the GALT.

In contrast to our earlier adoptive transfer experiments in which we immunized mice systemically, oral immunization with recombinant TG2-gluten fusion proteins together with CT failed to induce an anti-TG2 antibody response. This is likely due to degradation of the TG2-gluten antigen by gastric and luminal proteases.^14^ Immunoglobulins are relatively resistant to proteolytic degradation; although they can be partially cleaved by intestinal proteases, the resulting F(ab’)_2_ fragments retain antigen-binding capacity. We therefore adopted a strategy in which the gluten peptide containing the T-cell epitope is incorporated into an antibody specific for the idiotype of the 14E06 BCR,^18^ so-called Troybodies.^22^ This Troybody strategy effectively delivers T-cell epitopes to B cells.^23^ Here we integrated a deamidated omega-gliadin 18-mer peptide (*ω*18; QPEQPFPQ-PEQPFPWQPQ), harboring the DQ2.5-glia-*ω*2 T-cell epitope (underlined), into loop 6 of the C_H_1 domain of anti-14E06, generating the reagent *α*-14E06-*ω*18 Troy (Figure 1A). Importantly, the *α*-14E06-*ω*18 Troy bound equally well to 14E06 mAb as the *α*-14E06 control antibody with no gluten peptide introduced (Figure 1B). We next confirmed the ability of *α*-14E06-*ω*18 Troybody to load A20 B cells engineered to express the 14E06 BCR with the DQ2.5-glia-*ω*2 T-cell epitope for activation of gluten-specific CD4^+^ T cells. A20 B cells expressing the 14E06 BCR in combination with HLA-DQ2.5 were able to stimulate DQ2.5-glia-*ω*2-reactive CD4^+^ T cells, whereas A20 B cells expressing the control BCR 693–2-F02 were unable to induce T-cell proliferation (Figure 1C and D). As a control, both TG2-specific and nonspecific B cells induced T-cell proliferation when incubated with the free deamidated *ω*18 gliadin peptide (Supplementary Figure 2). These results demonstrate that the *α*-14E06-*ω*18 Troybody facilitates the interaction of TG2-specific 14E06 B cells with gluten-specific CD4^+^ T cells.

### Oral Immunization With mIgGI *α*-14E06-*ω*18 Troy and CT Induces CT-specific and TG2-specific IgA and IgG in Serum

To develop a model for the study of TG2-specific effector B cells in the gut, we transferred TG2-specific B cells isolated from *Tgm2*^−/−^ HLA-DQ2.5^+^ 14E06 KI mice along with gluten-specific T cells isolated from T-cell receptor-glia-*ω*2 transgenic mice^15^ to HLA-DQ2.5 KI recipient mice^16^ that were intragastrically immunized with *α*-14E06-*ω*18 Troy and CT, *α*-14E06 control antibody and CT, or CT alone (Figure 2A). Mice immunized with *α*-14E06-*ω*18 Troy and CT developed serum anti-TG2 antibodies that were not detected in mice immunized with CT alone, or in mice immunized with CT and the *α*-14E06 control antibody that lacks the T-cell epitope, indicating that the formation of anti-TG2 autoantibodies is dependent on help from gluten-specific T cells (Figure 2B).

We detected IgA anti-TG2 in the stool of most *α*-14E06-*ω*18 Troy-immunized mice (Figure 2C), and TG2-specific IgA plasma cells were present in the small intestinal lamina propria as detected by immunohistochemistry and ELISpot (Figure 2D and E). The number of anti-TG2 IgA-producing plasma cells in the small intestine correlated with anti-TG2 IgA levels in stool samples (Supplementary Figure 3A). Notably, anti-TG2 responses were similar between *Tgm2*^+/+^ and *Tgm2*^−/−^ HLA-DQ2.5 KI recipient mice immunized with *α*-14E06-*ω*18 Troy and CT (Supplementary Figure 3B and C), further supporting our earlier observation that TG2-specific B cells are not tolerized in GALT, also in adoptive transfer experiments. In parallel with anti-TG2 responses, mice developed anti-CT responses as indicated by IgG and IgA antibodies in serum, anti-CT IgA in stool, and CT-specific IgA plasma cells in the gut (Supplementary Figure 4A–C). These results align with our recent findings that anti-CT antibodies are induced in HLA-DQ2.5 KI mice devoid of murine major histocompatibility complex (MHC) class II molecules, albeit at reduced levels in comparison with wild-type mice.^24^

### TG2-specific B Cells Are Present in PP and Mesenteric Lymph Node

We next analyzed TG2-specific B-cell responses at inductive sites for the mucosal immune system. Adoptively transferred TG2-specific 14E06 B cells were expanded in both PP and mesenteric lymph node (MLN) after 2 and 3 rounds of immunization with *α*-14E06-*ω*18 Troy (Figure 3A, Supplementary Figure 5A and B). In line with our observations of anti-TG2 IgA plasma cells in lamina propria, there was substantial variation between mice in the magnitude of the B-cell response in both PPs and MLNs. In mice with increased numbers of TG2-specific B cells in PPs and MLNs, most transferred 14E06 B cells were IgD negative whereas IgD^−^ 14E06 B cells were not detected in mice immunized with *α*-14E06 control antibody, or with CT alone. Transferred gluten-specific CD4^+^ T cells were detected at increased frequencies in MLN of *α*-14E06-*ω*18 Troy-immunized mice (Figure 3B, Supplementary Figure 5C). A substantial proportion of these cells had adopted an activated CXCR5^hi^ programmed cell death protein 1 (PD-1)^hi^ T follicular helper (T_FH_)-cell phenotype (Figure 3C). The activated (IgD^−^) TG2-specific B cells in MLN stained for markers suggesting a germinal center-derived (CD95^+^ GL7^+^) phenotype (Figure 3D). In PPs of *α*-14E06-*ω*18 Troy-immunized mice, most class-switched 14E06 B cells were CD38^−^ CD95^+^. In contrast to the activated TG2-specific B cells in MLN, GL7 was expressed on only a subset of IgD^−^ CD38^−^ CD95^+^ 14E06^+^ B cells in PPs, which could be in line with previous observations that the GL7 phenotype in PPs is reversible.^12^ As flow cytometry does not disclose the anatomic location of the activated TG2-specific B cells in PPs, we also analyzed frozen tissue sections by immunohistochemical staining. Staining of tissue sections for Ephrin-B1 that defines germinal centers, revealed the presence of 14E06^+^ B cells in this area but notably also in other areas (Figure 3F). Thus, although 14E06^+^ B cells appear to be engaged in germinal center reactions, extrafollicular reactions may also take place during the *α*-14E06-*ω*18 Troy-induced immune response. Importantly, we also observed the presence of 14E06^+^ B cells in areas stained for receptor activator of NF-κB ligand (RANKL) that defines SED areas.

### TG2-specific B Cells in PP SED Take Up TG2 Antigen Directly From the Gut Lumen

IgD^−^ B cells in SED have been previously shown to have an antigen sampling function.^12^ Having established a mouse model in which mice have anti-TG2 effector B cells in the gut and GALT, we next questioned whether TG2-specific B cells in the SED area of PPs could take up TG2 antigen directly from the gut lumen. Thereto, we adopted a ligated loop protocol of the small intestine^12^ in HLA-DQ2.5 KI mice that received naive TG2-specific B cells and gluten-specific CD4^+^ T cells by adoptive transfer and were immunized twice with *α*-14E06-*ω*18 Troybody (Figure 4A). We made use of the sulfo-Cy5-labeled TG2 inhibitor HB230, which is a small gluten-peptide mimetic that irreversibly binds to the TG2 active site.^20^ Recombinant murine TG2 was incubated with HB230 in vitro at a 1:3 molar ratio before injection into ligated loops of the small intestine containing 1 or 2 PPs. After 2 hours, loops were removed, and PPs were fixed and frozen for cryosectioning. The injected antigen was detected in the SED areas but not deeper in the PP tissue, and HB230 signal was detected in 14E06^+^ B cells but not in 14E06^−^ endogenous B cells (Figure 4B–D). Importantly, no signal was detected when recombinant TG2 incubated with the negative control analog HB258 that is identical to HB230 but lacks the electrophilic warhead required for binding to the TG2 active site cysteine, was injected into ligated loops. These results indicate that the TG2-specific BCR was required for antigen binding. Of note, when HB230 was injected into ligated loops to probe for catalytically active endogenous enzyme in the lumen, we did not detect any Cy5 signal in the PPs.

## Discussion

The crosstalk between TG2-specific B cells and gluten-specific CD4^+^ T cells through the formation of TG2-gluten complexes is increasingly recognized as a central step in the pathogenesis of celiac disease. TG2-specific IgA plasma cells, being abundant in the small intestinal lamina propria of untreated celiac patients, disappear quickly when patients are on a gluten-free diet,^25^ suggesting that the continuous formation of TG2-gluten complexes that fuel T-cell-B-cell interactions are required to sustain the anti-TG2 IgA response in celiac disease. We have proposed that TG2 derived from shed enterocytes could form complexes with gluten peptides in the gut lumen and stimulate TG2-specific B cells in GALT.^10^ Although we have previously found that the TG2 enzyme in enterocyte lysates is catalytically active and able to form complexes with gluten that are recognized by TG2-specific B cells, allowing for the presentation of deamidated gluten epitopes to gluten-specific T cells in vitro,^10^ in vivo models are needed to assess whether TG2-gluten complexes can be formed in the gut lumen and taken up into GALT.

In this work, we have developed a model that allows for the study of T-cell dependent TG2-specific B-cell responses in GALT. By using Troybodies to deliver DGPs for antigen presentation by TG2-specific B cells, we demonstrate that HLA-DQ2.5 KI mice, on oral immunization, develop anti-TG2 gut humoral responses in addition to gluten-specific T-cell memory. This model mirrors the adaptive immune profile observed in patients with established celiac disease. TG2-specific B cells in the SED area of PPs of immunized mice were able to bind recombinant TG2 antigen complexed with the fluorescent TG2 inhibitor HB230 that was delivered into the gut lumen in ligated loops of the small intestine. However, when HB230, designed as a small peptide based on the TG2 recognition site of its gluten peptide substrate PQLPY, was injected into ligated loops to probe for catalytically active endogenous enzyme in the lumen, we did not detect any Cy5 signal in the PPs. This observation could be explained by limitations of the sensitivity of our approach and a high Cy5 signal being necessary to detect uptake of luminal antigen by immunohistochemistry.

The amount of TG2 that is released in the lumen from shed enterocytes depends on the turnover rate of enterocytes, as well as on the amount of TG2 per cell. The epithelial turnover rate is drastically increased in untreated celiac disease, reducing the normal enterocyte lifespan from 3 to 5 days to about 24 hours.^26^ In addition, TG2 expression in enterocytes of patients with active celiac disease is elevated.^27^ Thus, it is likely that in active celiac disease, increased amounts of TG2 are present in the gut lumen. Elevated levels of TG2 in the lumen also can be achieved by other conditions that (transiently) increase the epithelial turnover rate, such as common gastrointestinal infections. Rotavirus infection in mice, for example, has been shown to result in increased epithelial turnover and increased enterocyte shedding.^28,29^ Common gastrointestinal viral infections, including rotavirus, have been associated as contributing factors for the onset of celiac disease, although the mechanisms by which they could trigger disease development are not clear.^30^ An increase in luminal TG2 could be an important stepping-stone for the amplification of the gluten-specific T-cell response as it would generate a larger number of DGPs that can be presented on disease-associated HLA-DQ molecules to the T cells. This idea would fit with a threshold model for the development of celiac disease, in which the efficiency of gluten presentation to CD4^+^ T cells determines the likelihood of developing celiac disease.^31^

Our finding that TG2-specific B cells in PPs take up luminally perfused TG2 directly from the gut lumen confirms a previous study that demonstrated that activated antigen-specific B cells in PPs closely interact with M cells in the follicle-associated epithelium, bind luminal antigens, and migrate to the germinal center, proposing a novel pathway for the transport of antigens from the SED to the germinal center.^12^ Our findings are relevant for celiac disease where TG2-specific B cells in GALT likely represent the main antigen-presenting cells during active disease. TG2-specific B cells in celiac disease bind a few restricted conformational epitopes in the N-terminal domain of TG2.^32^ The B cells thus presumably bind active enzymes that can react with gluten peptides even when bound to the BCR.^8,33^ Thus, TG2-specific B cells located in the SED are ideally located on the front line to take up both TG2 released from shed enterocytes and gluten from the lumen. This allows for quick encounter of intact luminal antigens, which will be important given that TG2 in the gut lumen is prone to degradation. In human PPs, the presence of B cells in the SED in close association to the follicle-associated epithelium has also been demonstrated.^34,35^ Whether TG2-specific B cells are localized close to the gut lumen in the PPs of patients with celiac disease needs to be addressed in future studies.

A role for DGP-specific B cells in presenting gluten antigen to T cells could not be evaluated in our current model. DGP-specific B cells have been shown to efficiently activate gluten-specific CD4^+^ T cells by uptake and presentation of TG2-gluten complexes,^36^ suggesting that they could also acquire luminal TG2-gluten antigens in the same way as we show for TG2-specific B cells. However, the much higher frequency of TG2-specific compared with DGP-specific IgA plasma cells in celiac lesions indicates that antigen presentation to gluten-specific CD4^+^ T cells within GALT is more likely mediated by TG2-specific B cells. Furthermore, the epitopes recognized by DGP-specific B cells overlap with or are often adjacent to gluten T-cell epitopes,^37^ implying that DGP-specific B cells in patients may bind soluble peptide antigens rather then TG2-gluten complexes. Because gluten peptides can distribute systemically, B-cell responses to gluten may originate both at GALT and MLN but also at sites distant from the gut. This is reflected by the strong anti-DGP IgG response in serum alongside anti-DGP IgA in patients with celiac disease, whereas the anti-TG2 response is predominantly IgA.^38^

Growing evidence supports the role of B cells as antigen-presenting cells, not least because the specificity of B-cell receptors allow for efficient sampling and uptake of antigen for MHC II-mediated presentation.^39^ This evidence for the key role of B cells is not unique to celiac disease, similar evidence is accumulating also in numerous other autoimmune diseases such as rheumatoid arthritis, type 1 diabetes, and multiple sclerosis.^40^ Consequently, targeting B cells for depletion has become one of the most promising therapeutic strategies for treating autoimmune diseases.^40–42^ By the time patients receive treatment, their disease is chronic with clonally expanded antigen-specific B cells and the presence of memory B cells and plasma cells. It is therefore plausible that other antigen-presenting cells, such as dendritic cells, play an important role in the initial priming of T cells, partly through production of inflammatory cytokines. Nevertheless, increasing evidence indicates that antigen presentation by B cells also may be fundamental to disease initiation across multiple autoimmune disorders.^43–46^

B-cell-directed therapies have not yet been explored for the treatment of celiac disease, but they might well be pursued in the future given their success for the other autoimmune diseases. Another promising strategy to disrupt the crosstalk between TG2-specific B cells and gluten-specific T cells is inhibition of the TG2 enzyme. A recent placebo-controlled phase 2 clinical trial demonstrated the potential for this as a therapy, as oral administration of the reversible TG2 inhibitor TAK-227/ZED-1227 prevented gluten-induced intestinal remodeling and disease relapse during a 6-week gluten challenge.^47^ Transcriptomic analysis of gut biopsies from trial participants suggests that TAK-227/ZED-1227 attenuates gluten-driven T-cell immune reactions,^48^ possibly by interfering with T-cell-B-cell interactions. Notably, TAK-227/ZED-1227 has poor systemic absorption,^49^ indicating that its clinical efficacy may well result from inhibition of TG2 activity within the gut lumen. We anticipate that further development of the model we describe here will provide a valuable foundation for the development of improved TG2 inhibitors and novel therapeutic strategies for celiac disease.

## Supplementary Material

1

Note: To access the supplementary material accompanying this article, visit the online version of *Gastroenterology* at www.gastrojournal.org, and at https://doi.org/10.1053/j.gastro.2025.06.021.

## Figures and Tables

**Figure 1.**
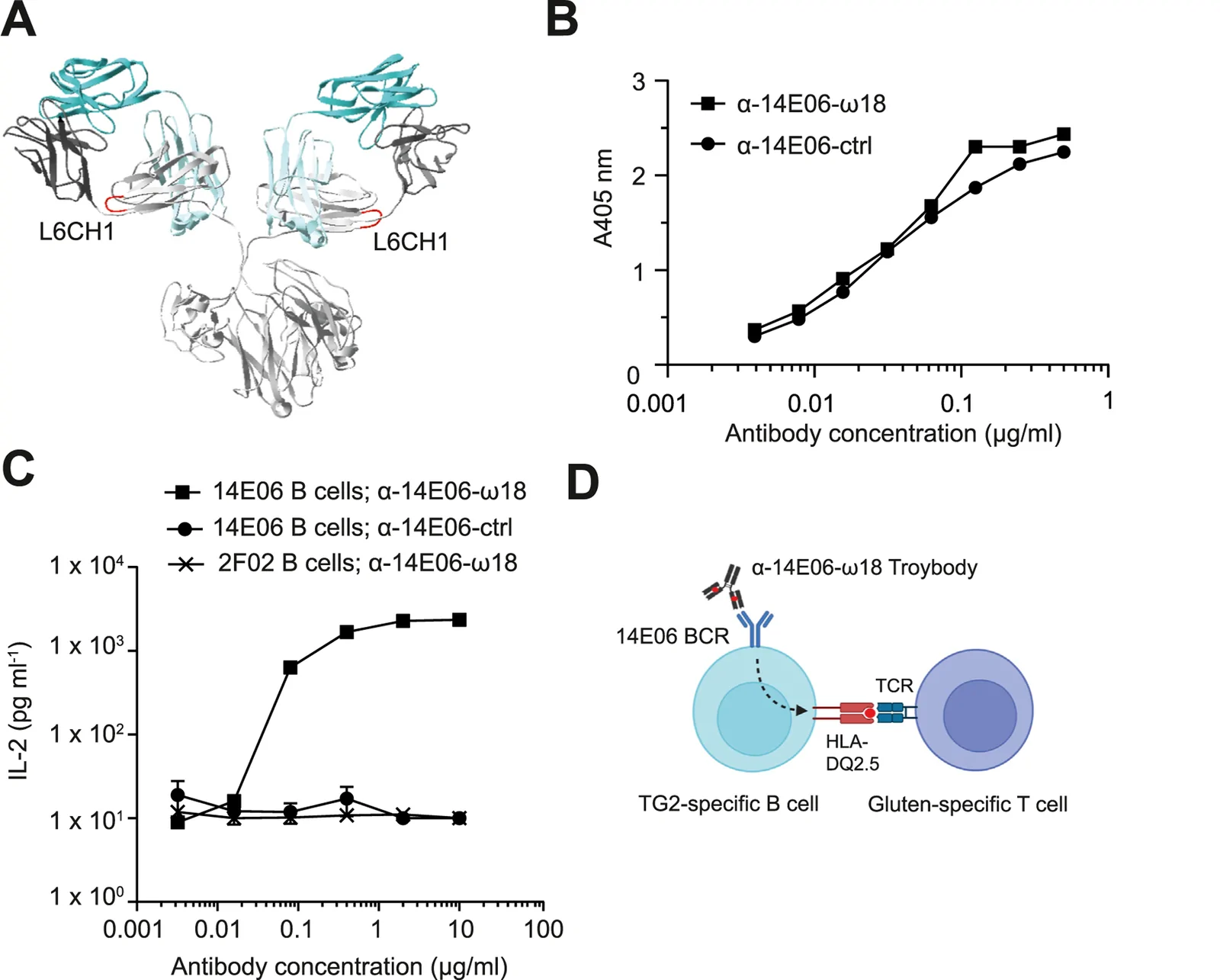
Troybody targeting the 14E06 anti-TG2 BCR is efficiently taken up by specific B cells and delivers a gluten T-cell epitope for activation of guten-specific T cells. (*A*) Ribbon illustration of an mIgG1 indicating the position of loop 6 between connecting *β* strands of the constant heavy domain CH1, indicated L6CH1 (*red*). The *ω*18-mer gluten peptide was inserted by exchange of the central loop amino acids. IgG heavy and light chain are depicted in *gray* and *cyan,* respectively. The figure was prepared in PyMOL using PDB ID 1IGY. (*B*) Antibody binding of mIgG1 *α*-14E06-*ω*18 L6CH1 Troybody and the mIgG1 anti-14E06 control antibody toward the TG2-reactive recombinant hmAb 14E06 was confirmed by ELISA. Plates were coated with hmAb 14E06 expressed as IgA1 and detected with goat-anti-mouse IgG. Symbols represent mean absorbance A405 values of assay duplicates. (*C*) Activation of BW58 T cells transduced to express the DQ2.5-glia-*ω*2-reactive TCR measured as release of interleukin (IL)-2 after co-culture with HLA-DQ2.5-expressing A20 B cells in the presence of *α*-14E06-*ω*18 Troybody or *α*-14E06-control antibody. TG2-reactive B cells expressing the 14E06 BCR, but not nonspecific B cells expressing the 2-F02 BCR, were able to present deamidated gluten peptide to gluten-specific CD4^+^ T cells after incubation with *α*-14E06-*ω*18 Troybody. Symbols represent mean IL-2 concentration with indication of standard deviation based on culture triplicates. Results from 1 of 2 experiments are shown. (*D*) Schematic representation of *α*-14E06-*ω*18 Troybody-facilitated collaboration between 14E06 BCR-expressing TG2-specific B cells and gluten-specific T cells. Troybody binding to the 14E06 BCR results in internalization and release of the deamidated gluten peptide (in *red*) for presentation on HLA-DQ2.5 to gluten-specific CD4^+^ T cells.

**Figure 2.**
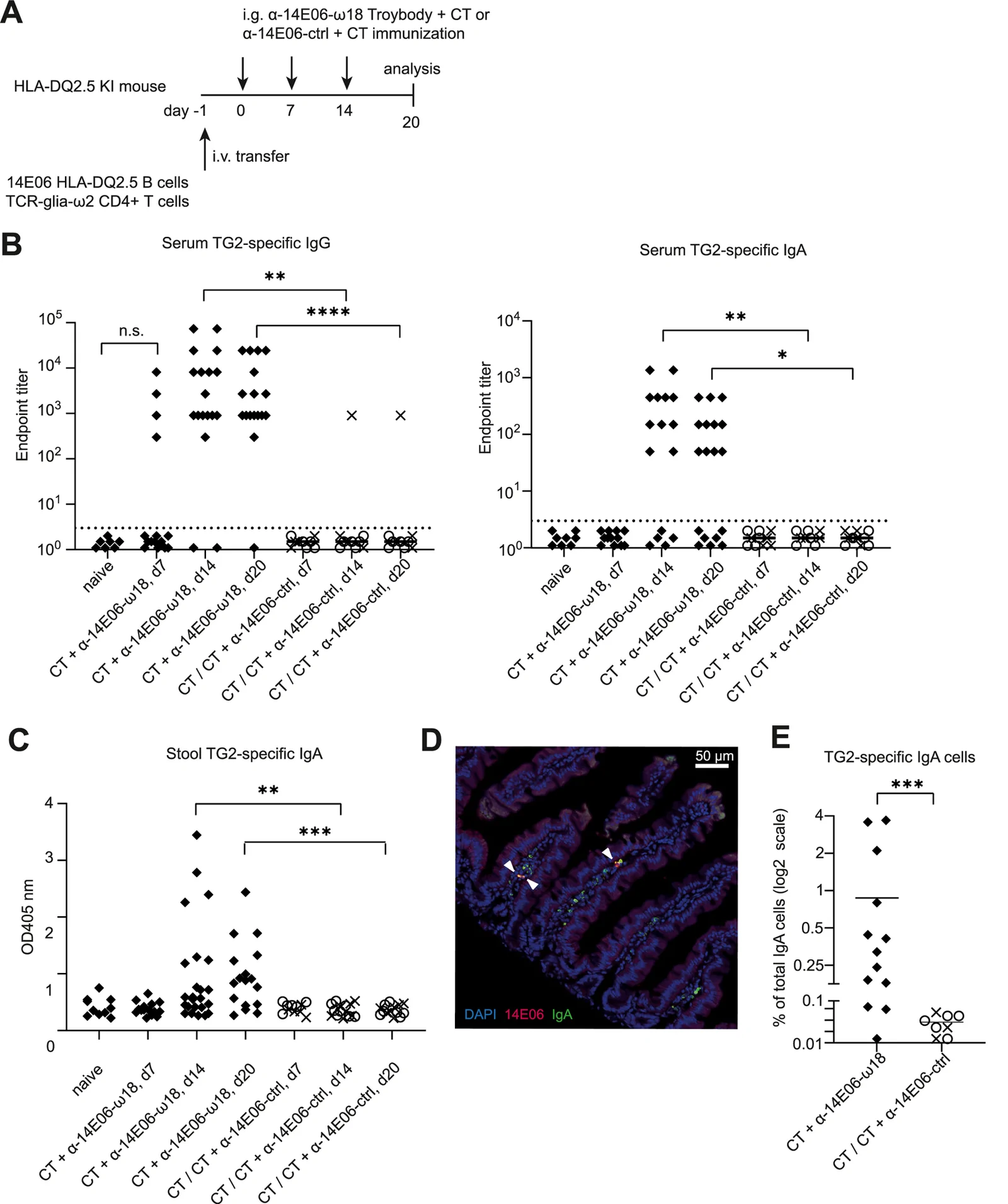
Oral immunization with mIgG1 *α*-14E06-*ω*18 Troy and CT induces TG2-specific IgA and IgG in serum. (*A*) Schematic representation of the adoptive transfer model and intragastric (i.g.) immunization with mIgG1 *α*-14E06-*ω*18 Troy and CT, or of mIgG1 *α*-14E06-ctrl and CT or CT alone. Serum and stool samples were collected on days 0, 7, 14, and 20. (*B*) ELISA endpoint titer quantification of TG2-specific serum IgG and IgA antibodies in HLA-DQ2.5 KI mice with adoptively transferred TG2-specific B cells and gluten-specific CD4^+^ T cells immunized i.g. with *α*-14E06-*ω*18 Troy and CT (*diamonds*), *α*-14E06-ctrl and CT (*crosses*), or CT alone (*circles*). ELISA endpoint titer was defined as the reciprocal of the last dilution giving an absorbance above the cutoff. (*C*) TG2-specific IgA antibodies in stool (diluted 1:2) of mice orally immunized with *α*-14E06-*ω*18 Troy and CT (*diamonds*), *α*-14E06-ctrl and CT (*crosses*), or CT alone (*circles*). (*D*) Representative image showing the small intestine of a mouse immunized 3 times with mIgG1 *α*-14E06-*ω*18 Troy and CT. Tissue section was stained with anti-IgA (*green*), anti-14E06 (*red*), and 4’,6-diamidino-2-phenylindole (DAPI) (*blue). (E*) ELISpot quantification of TG2-specific IgA plasma cells per total IgA plasma cells in the small intestinal lamina propria. (*B,C,E*) Data are pooled from 4 independent experiments. Error bars indicate standard deviation. Median of each group was compared between groups with Mann-Whitney *U* test. *, **, ***, and **** indicate significant differences with *P* < .05, *P* < .005, *P* < .0005, and *P* < .0001, respectively. n.s., not significant.

**Figure 3.**
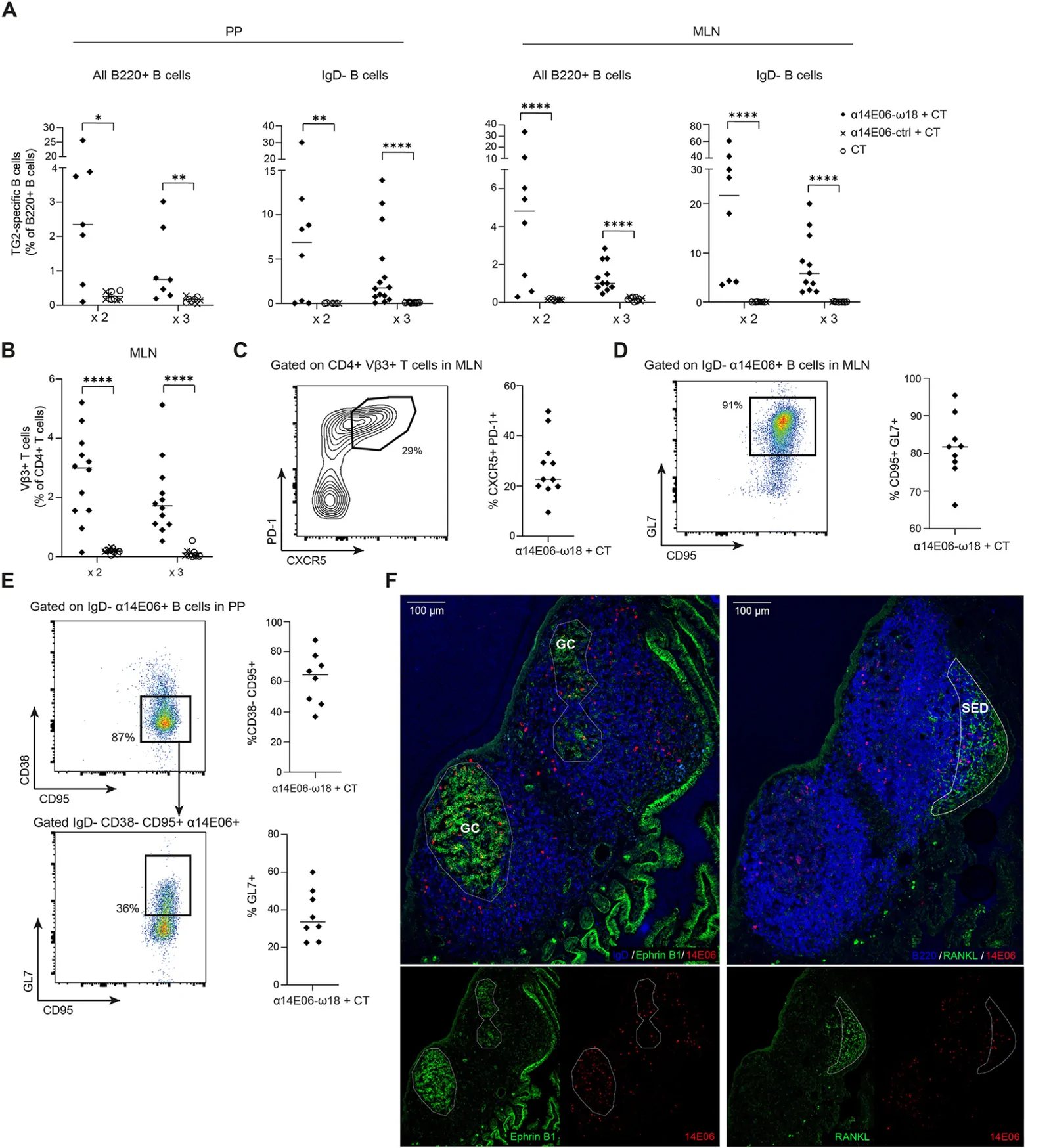
Activated TG2-specific B cells are present in MLNs and PPs following immunization with *α*-14E06-*ω*18 Troy and locate to the germinal center and sub-epithelial dome in PPs. (*A*) Frequency of adoptively transferred TG2-specific B cells in PPs and MLNs of CD45.1 HLA-DQ2.5 KI recipient mice immunized 2 or 3 times with *α*-14E06-*ω*18 Troy and CT (*diamonds*), *α*-14E06-ctrl and CT (crosses), or CT alone (*circles*). The experimental model is depicted in Figure 2A, and mice were analyzed on day 14 in case of 2 immunizations or on day 20 in case of 3 immunizations. Data are presented as percentages of CD45.2^+^ and *α*-14E06^+^ B220^+^ B cells of either the total B220^+^ population or of class-switched IgD^−^ B cells in PPs and MLNs. (*B*) Frequency of adoptively transferred transgenic (human TCR V*β*3^+^) gluten-specific CD4^+^ T cells as percentage of total CD4^+^ in MLNs of mice immunized 2 or 3 times with *α*-14E06-*ω*18 Troy and CT (*diamonds*), *α*-14E06-ctrl and CT (crosses), or CT alone (*circles*). (*C*) Representative flow cytometry contour plot and percentages of PD-1^+^ CXCR5^+^ T_FH_ cells among transgenic gluten-specific CD4^+^ V*β*3^+^ T cells in MLNs on day 14 of mice immunized twice with *α*-14E06-*ω*18 Troy. (*D*) Representative flow cytometry plot and percentages showing germinal center (CD95^+^ GL7^+^) B cells in the adoptively transferred IgD^−^ CD45.2^+^ *α*-14E06^+^ B cells in MLNs on day 14 of mice immunized twice with *α*-14E06-*ω*18 Troy. (*E*) Representative flow plots and percentages of CD38^−^ CD95^+^ and GL7^+^ B cells in the adoptively transferred IgD^−^ CD45.2^+^ *α*-14E06^+^ B cells in PPs on day 14 of mice immunized twice with *α*-14E06-*ω*18 Troy. (*F*) Representative microscopy images of PP tissue sections following 2 oral immunizations with *α*-14E06-*ω*18 Troy and CT. The image on the *left* shows TG2-specific B cells (14E06, *red*) in the germinal center (GC) identified by absence of staining for IgD (*blue*) combined with Ephrin B1 (*green*) staining. The image on the *right* shows B220^+^ (*blue*) and 14E06^+^ (*red*) B cells with the SED area of the PP visualized with staining for RANKL (*green*). Data are combined from 2 or 3 independent experiments. Median of each group was compared between groups with Mann-Whitney *U* test. Error bars indicate standard deviation. *, **, and **** indicate significant differences with *P* < .05, *P* < .005, and *P* < .0001, respectively.

**Figure 4.**
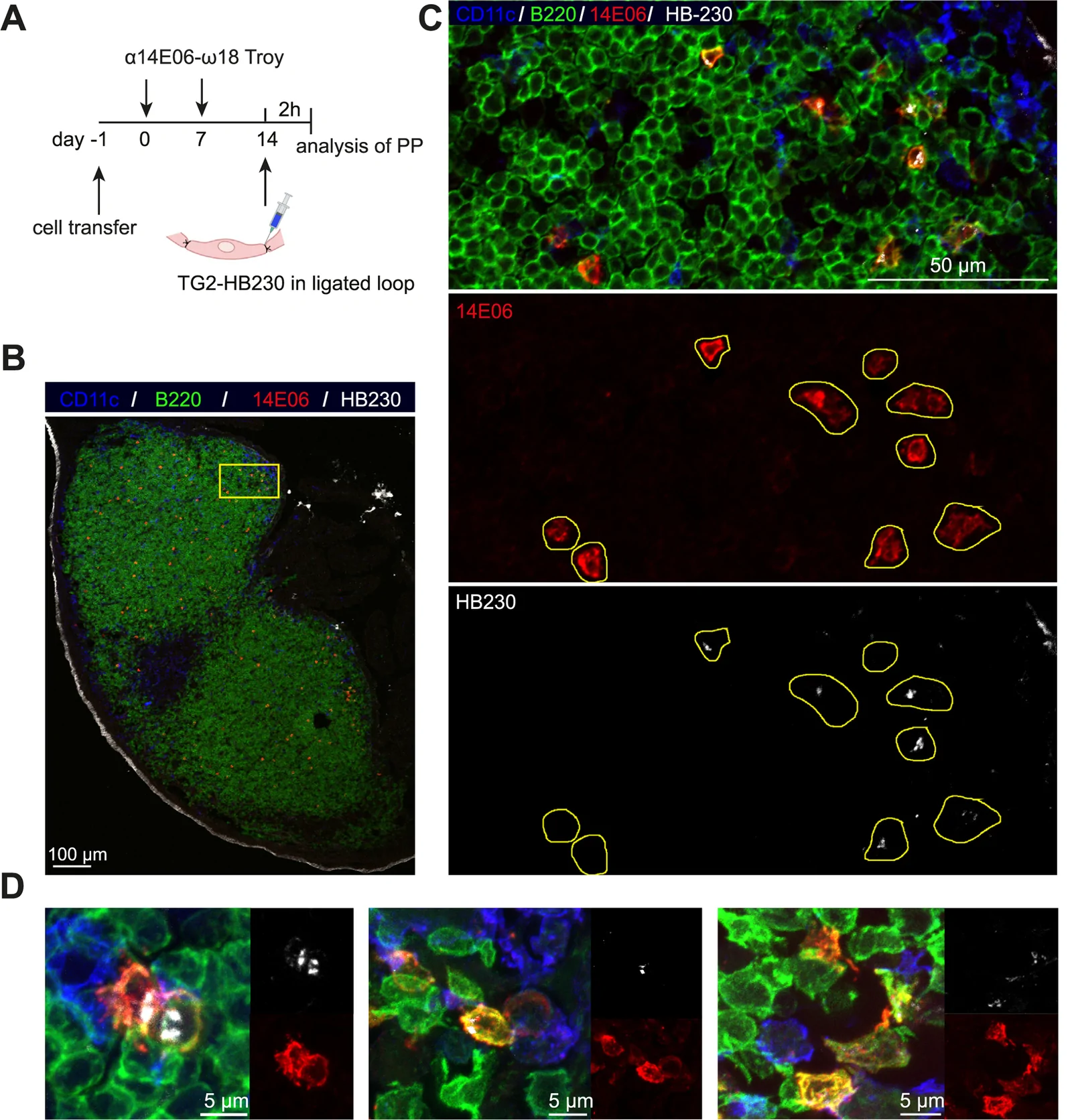
TG2-specific B cells in SED take up antigen directly from the gut lumen. (*A*) Schematic representation of the ligated loop model. HLA-DQ2 KI mice received naive TG2-specific B cells and gluten-specific CD4^+^ T cells by adoptive cell transfer, and were immunized twice with mIgG1 *α*-14E06-*ω*18 Troybody and CT. Uptake of TG2-HB-230 from the gut lumen was studied 1 week after the second immunization in a ligated loop model. (*B*) Representative microscopy overview image showing a PP 2 hours after injection of TG2-HB-230 in the gut lumen. (*C*) Close-ups of the SED area indicated in (*B*) show HB-230 (*white*) associated with TG2-specific B cells (14E06, *red*). B220^+^ B cells are shown in *green,* CD11c^+^ dendritic cells in *blue.* (*D*) Maximum-intensity projections of confocal Z-stacks showing accumulation of TG2-HB-230 antigen (*white*) taken up by TG2-specific (14E06, *red*) B cells (B220, *green*). CD11c^+^ dendritic cells are shown in *blue.* The data shown are representative of 4 mice (2 *Tgm2*^+/+^ and 2 *Tgm2*^−/−^ HLA-DQ2.5 KI mice), where at least 1 PP is analyzed for each mouse.

## Data Availability

Reagents can be made available under MTAs. HB230 is now commercially available from Bio-Techne (catalog number 8831).

## WHAT YOU NEED TO KNOW

### BACKGROUND AND CONTEXT

The gut lumen has been proposed to be the location where pathogenic TG2, derived from shed enterocytes, encounters gluten peptides in celiac disease, thereby leading to uptake of TG2-gluten complexes in gut-associated lymphoid tissue and allowing for local collaboration of TG2-specific B cells and gluten-specific T cells.

### NEW FINDINGS

By developing a mouse model, we demonstrate that transglutaminase 2-specific B cells in the Peyer’s patches can sample recombinant TG2 directly from the gut lumen.

### LIMITATIONS

Whether enzyme-substrate complexes formed between endogenous transglutaminase 2 and gluten can be taken up by transglutaminase 2–specific B cells in Peyer’s patches remains to be determined. Furthermore, the contribution of other antigen-presenting cell subsets in the uptake and presentation of gluten antigens was not addressed in the model.

### CLINICAL RESEARCH RELEVANCE

The work provides further evidence for the gut lumen as the site where transglutaminase 2 acts on gluten peptides. We anticipate that the model will give foundation for the development and testing of new therapies for celiac disease, including improved transglutaminase 2 inhibitors and strategies for the depletion of autoreactive B cells.

### BASIC RESEARCH RELEVANCE

The findings support the notion that luminal transglutaminase 2 drives interactions between transglutaminase 2-specific B cells and gluten-specific T cells in gut-associated lymphoid tissue, and they offer a valuable preclinical tool to dissect these pathogenic T-cell-B-cell interactions.

## Abbreviations used in this paper:

BCR: B-cell receptor
CT: cholera toxin
DGP: deamidated gluten peptide
ELISA: enzyme-linked immunosorbent assay
ELISpot: enzyme-linked immunosorbent spot
GALT: gut-associated lymphoid tissue
HLA: associated human leukocyte antigen
Ig: immunoglobulin
KI: knockin
M cell: microfold cell
MLN: mesenteric lymph node
PBS: phosphate-buffered saline
PP: Peyer’s patch
TCR: T-cell receptor
TG2: transglutaminase 2
SED: sub-epithelial dome
