# Supplementary material for: Peyer’s Patch B Cells Sample Transglutaminase-Gluten Complexes and Drive Celiac Disease Autoimmunity
Source: Gastroenterology. Author manuscript; Available in PMC 2026 Nov 11. (PMC13752806; doi:10.1053/j.gastro.2025.06.021)
Supplement: Supplementary file 1 [file NIHMS2169011-supplement-1.pdf]

Supplementary Material and Methods

Anti-14E06 antibody and anti-14E06-ω18 Troybody production

Anti-14E06 Troybodies were expressed as mIgG1 and mIgG2b. Synthetic gene fragments encoding V_H and V_L of the anti-14E06 monoclonal antibody 2G9[1] were obtained from GenScript. To generate the mIgG1 anti-14E06-ω18 Troybody and mIgG1 anti-14E06 control antibody, The V_H gene fragment was cloned into EcoRI and AfeI sites of the pFUSEss-CHIg-mG1 plasmid (InvivoGen). For the mIgG1 anti-14E06-ω18 Troybody, the 18mer ω-gliadin peptide sequence QPEQPFPQPEQPFPWQPQ containing the DQ2.5-glia-ω2 T-cell epitope (underlined) was inserted into loop 6 of C_H1 domain between BamHI and PmlI sites, replacing the four native amino acids KPSN as previously described[2]. For both the Troybody and the control antibody the V_L gene fragment was cloned into EcoRI/BstAPI sites of the pFUSE2-CLIg-mk plasmid (InvivoGen). For generation of the mIgG2b anti-14E06-ω18 Troybody, a synthetic gene fragment encoding V_H and the 18mer ω-gliadin peptide sequence introduced in loop 6 of the C_H1 domain was obtained from GenScript and cloned into the pLNOH2-Cγ2b expression vector[3] between BsmI and BstEII sites. To generate the light chain vector, a gene fragment containing the V_L and mouse Cκ was cloned into BsmI/BamHI sites of the pLNOκ plasmid[4]. The mIgG1 anti-14E06 antibodies and Troybodies were expressed in Expi293-F cells by transient transfection using 1 mg/ml polyethyleneimine (23966-1, Polysciences). The transfected cells were cultured in Expi293F Expression medium (Gibco) in shaker flasks at 37 °C, 8% CO_2 for 6 days before the supernatant was harvested. Secreted antibodies were purified using CaptureSelect LC-kappa (murine) Affinity Matrix resin (191315005, Thermo Fisher Scientific) applied to a Tricorn 5 column and eluted using 0.1 M Glycine-HCl pH 2.5. Fractions were immediately neutralized by addition of Tris-HCl pH 9.0, before the antibodies were dialyzed overnight to PBS using 20.000 MWCO Slize-A-Lyzer dialysis cassettes (Thermo Scientific). As higher yields were achieved with the production of the mIgG1 anti-14E06-ω18 Troybody, this isotype was used for most experiments.

Cell lines

Transduced A20 mouse lymphoma B cells expressing HLA-DQ2.5 and the TG2-specific IgD BCR 679-14-E06 or non-specific BCR 693-2-F02[5] and transduced mouse BW58α-β T cells devoid of endogenous T-cell receptors expressing human CD4 and a DQ2.5-glia-ω2–reactive TCR[6] were previously described. Transfectant B cells (50,000) were incubated with indicated concentrations of anti-14E06-ω18 Troybody, α-14E06-control antibody or deamidated ω18 gliadin peptide (QPEQPFPQPEQPFPWQPQ, GenScript) in

RPMI 1640 supplemented with 10% (v/v) FCS for 4 hours in 96-well U-bottom plates at 37°C, 5% CO_2 before transfectant T cells (25,000) were added. After 18 hours, culture supernatants were collected and assayed for murine IL-2 production by ELISA.

Adoptive transfer and immunization

TG2-specific B cells were isolated from spleens of HLA-DQ2.5 14E06 KI mice. Single cell suspensions were prepared by carefully grinding and filtering spleens through a 70 µm diameter nylon mesh. After removal of erythrocytes by treatment with ammonium-chloride-potassium (ACK) lysis buffer, B cells were isolated using the Dynabeads Mouse CD43 kit (Invitrogen). The purity of isolated B cells, measured as the percentage of $B220^+$ cells by flow cytometry, was typically >95%. Gluten-specific CD4+ T cells were isolated from pooled lymph nodes and spleens of HLA-DQ2.5 TCR-glia-ω2 transgenic mice by negative selection using the EasySep Mouse $CD4^+$ T cell isolation kit (Stemcell Technologies). Homozygous CD45.1 HLA-DQ2.5 recipient mice received 1 x 10^6 TG2-specific B cells and 5 x 10^5 gluten-specific $CD4^+$ $Vß3^+$ T cells by intravenous injection in the lateral tail vein. The next day, mice were immunized intragastrically with 250-400 µg α-14E06-ω18 Troybody or anti-14E06 control antibody and/or 10 µg cholera toxin (100B, List Biological Laboratories) in phosphate-buffered saline (PBS) with 3% (w/v) $NaHCO_3$. Mice were fasted for 1 hour before oral immunization. Booster immunizations were given after 7 and 14 days.

ELISA

To assess binding of the mIgG1 anti-14E06-ω18 L6CH1 Troybody and the mIgG1 anti-14E06 control antibody to hmAb 14E06, 96-well Maxisorp ELISA plates (Nunc) were coated overnight with 4 µg/ml hmAb 14E06 expressed as IgA1[5] in 100 µl 0.05 M carbonate/bicarbonate buffer pH 9.6 overnight at 4°C. Plates were washed, subsequently blocked with 200 µl 2% bovine serum albumin in PBS 0.05% (v/v) Tween-20 (PBS-T) and incubated with indicated dilutions of mIgG1 anti-14E06-ω18 L6CH1 Troybody and mIgG1 anti-14E06 control for 2h at room temperature (RT). After washing, the plates were incubated with alkaline phosphatase (AP)-conjugated goat-anti-mouse IgG (Sigma A2429) diluted 1:5000 in PBS 0.05% (v/v) Tween-20 for 1h at room temperature, washed again, and incubated with 1 mg/ml phosphatase substrate (Sigma). Absorbance was measured in a plate reader (Multiskan ascent, ThemoFisher Scientific) at 405 nm.

For measurement of IL-2 in culture supernatants, wells were coated with 2 µg/ml rat-anti-mouse-IL-2 (clone JES6-5H4, Pharmingen) and detected with 1 µg/ml biotin-rat-anti-mouse-IL-2 (clone JES6-1A12,

Pharmingen). IL-2 concentrations were calculated from a standard curve generated using recombinant murine IL-2 (BioLegend).

For measurement of anti-TG2 and anti-CT antibodies in serum samples, mice were bled from the saphenous vein and blood was allowed to clot for 20-30 min at room temperature before centrifugation for 15 min at 2,000 *g*, 4°C. Supernatant was collected and stored at -20°C until assayed. Recombinant mouse TG2 was expressed in *E.coli* and incubated with the irreversible active site inhibitor DP3-3 (Ac-P(DON)LPF-NH2; Zedira) for immobilization in an open confirmation as previously described[7], and used for overnight coating of ELISA plates at 5 µg/ml in PBS. For detection of anti-CT antibodies, plates were incubated with 0.5 nmol/ml GM1-ganglioside (Merck) for 2h at RT followed by 0.5 µg/ml CT overnight at 4°C. Following washing and blocking, serum samples were serially three-fold diluted in PBS-T and incubated for 2h at RT. Bound antibodies were detected by incubation with AP-conjugated goat anti-mouse IgG or AP-conjugated goat anti-mouse IgA (1:1000, 1040-04, Southern Biotech) for 1h at RT before development with 1mg/ml phosphatase substrate. Endpoint titers were defined as reciprocal of the last dilution giving an absorbance above the detection cut-off. Detection cut-off was set as the mean absorbance plus ten standard deviations of six or eight blank well measurements.

For detection of anti-TG2 antibodies in feces stool pellets were snap-frozen in liquid N_2. Pellets were rehydrated with 1 ml PBS supplemented with cOmplete protease inhibitor cocktail (Roche) per 100 mg stool and homogenized using a wooden toothpick followed by mixing using a MixMate tube mixer (Eppendorf). Homogenized samples were centrifuged for 10 min at 8,000 *g*, 4°C and supernatant was stored at -70°C. Due to high background after coating with TG2, plates were coated with 5 µg/ml hIgG1 anti-14E06 antibody[1] for detection of TG2-specific antibodies. Otherwise the procedure for detection of serum antibodies was followed.

Isolation of lamina propria lymphocytes

Small intestines, after removal of PP, were cut open longitudinally and washed in Ca^{2+}- and Mg^{2+}-free Hank's balanced salt solution (HBSS, Gibco) supplemented with HEPES (10 mM). The intestine was cut into ~0.5 cm pieces and tissue pieces were incubated twice in Ca^{2+}- and Mg^{2+}-free HBSS containing 10 mM HEPES, 5 mM EDTA (Invitrogen), 5% (v/v) FCS, and 1 mM ditriothreitol (DTT, Sigma) for 15 min at 37°C under continuous rotation. EDTA was washed away by incubating in Ca^{2+}- and Mg^{2+}-free HBSS containing 10 mM HEPES and 5% FCS for 15 min at 37°C under continuous rotation. Single cell

suspensions were prepared by digestion of the tissue pieces with 0.5 mg/ml Collagenase VIII (Sigma) in HBSS containing 10 mM HEPES and 5% (v/v) FCS running program 37C_m_LPDK_1 on a gentleMACS™ Octo Dissociator with heaters (Miltenyi Biotec). Cells were washed with PBS 2% FCS (v/v), and filtered over a 100 µm MACS Smartstrainer (Miltenyi Biotec).

References

1. **Hoydahl LS, Nilssen NR**, Gunnarsen KS, et al. Multivalent pIX phage display selects for distinct and improved antibody properties. Sci Rep 2016;6:39066.
2. Hoydahl LS, Frigstad T, Rasmussen IB, et al. Antibody-mediated delivery of T-cell epitopes to antigen-presenting cells induce strong CD4 and CD8 T-cell responses. Vaccine 2021;39:1583-1592.
3. Eidem JK, Rasmussen IB, Lunde E, et al. Recombinant antibodies as carrier proteins for sub-unit vaccines: influence of mode of fusion on protein production and T-cell activation. J Immunol Methods 2000;245:119-31.
4. Norderhaug L, Olafsen T, Michaelsen TE, Sandlie I. Versatile vectors for transient and stable expression of recombinant antibody molecules in mammalian cells. J Immunol Methods 1997;204:77-87.
5. Di Niro R, Mesin L, Zheng NY, et al. High abundance of plasma cells secreting transglutaminase 2-specific IgA autoantibodies with limited somatic hypermutation in celiac disease intestinal lesions. Nat Med 2012;18:441-5.
6. **Snir O, Chen X, Gidoni M,** et al. Stereotyped antibody responses target posttranslationally modified gluten in celiac disease. JCI Insight 2017;2.
7. du Pre MF, Blazevski J, Dewan AE, et al. B cell tolerance and antibody production to the celiac disease autoantigen transglutaminase 2. J Exp Med 2020;217.

Author names in bold designate shared co-first authorship.

Supplementary Figures

Supplementary Figure 1:

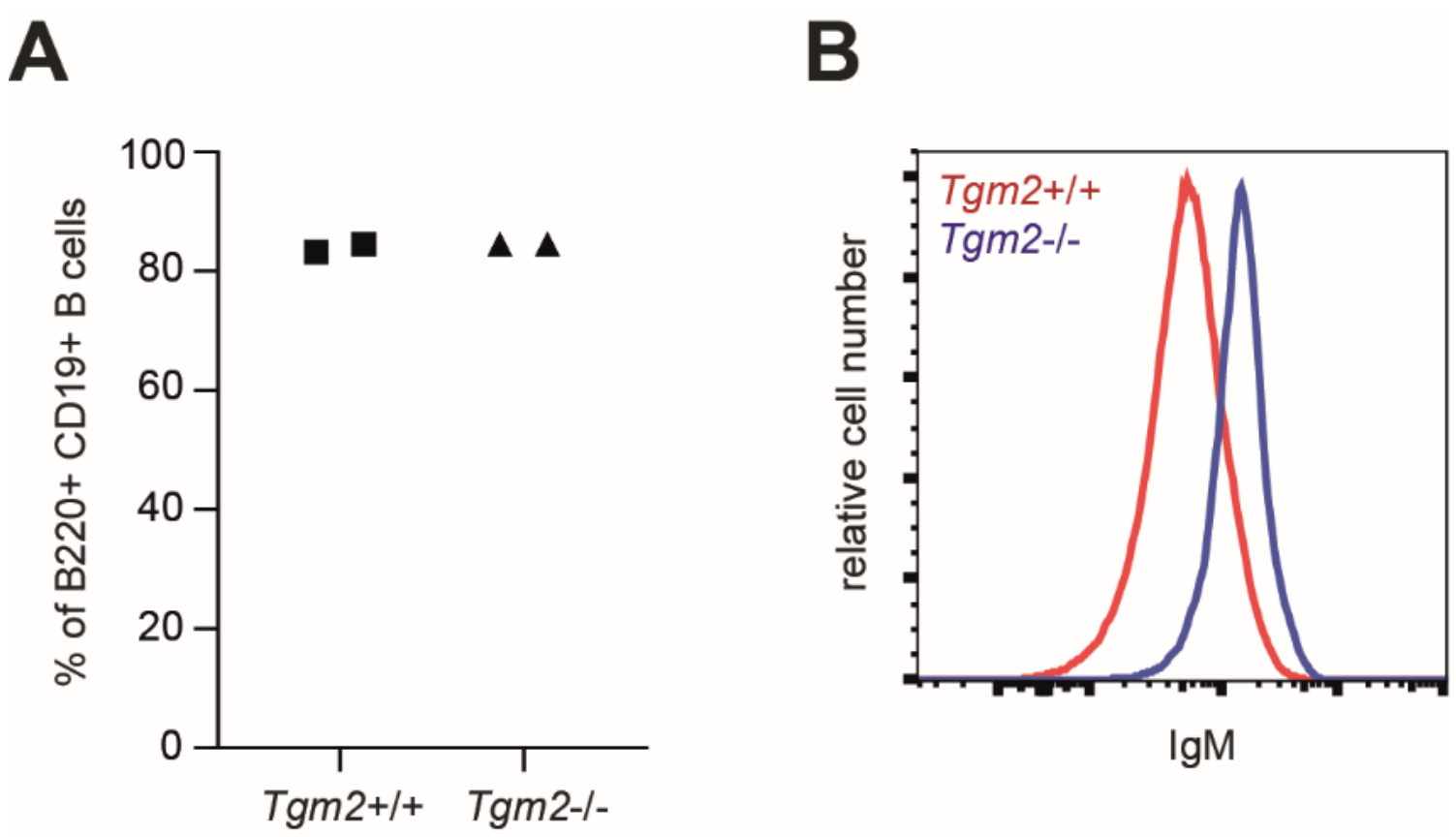

Supplementary Figure 1. Absence of B-cell tolerance induction in Peyer's patches. (*A*) Percentage of TG2-specific B cells as detected by anti-14E06 antibody staining in Peyer's patches of $Tgm2^{+/+}$ and $Tgm2^{-/-}$ 14E06 KI mice. Each data point represents pooled Peyer's patches from one mouse. (*B*) Histogram plot showing the expression of surface IgM; gated on $B220^{+}$ $CD19^{+}$ anti-$14E06^{+}$ B cells in Peyer's patches of $Tgm2^{+/+}$ and $Tgm2^{-/-}$ 14E06 KI mice.

Supplementary Figure 2:

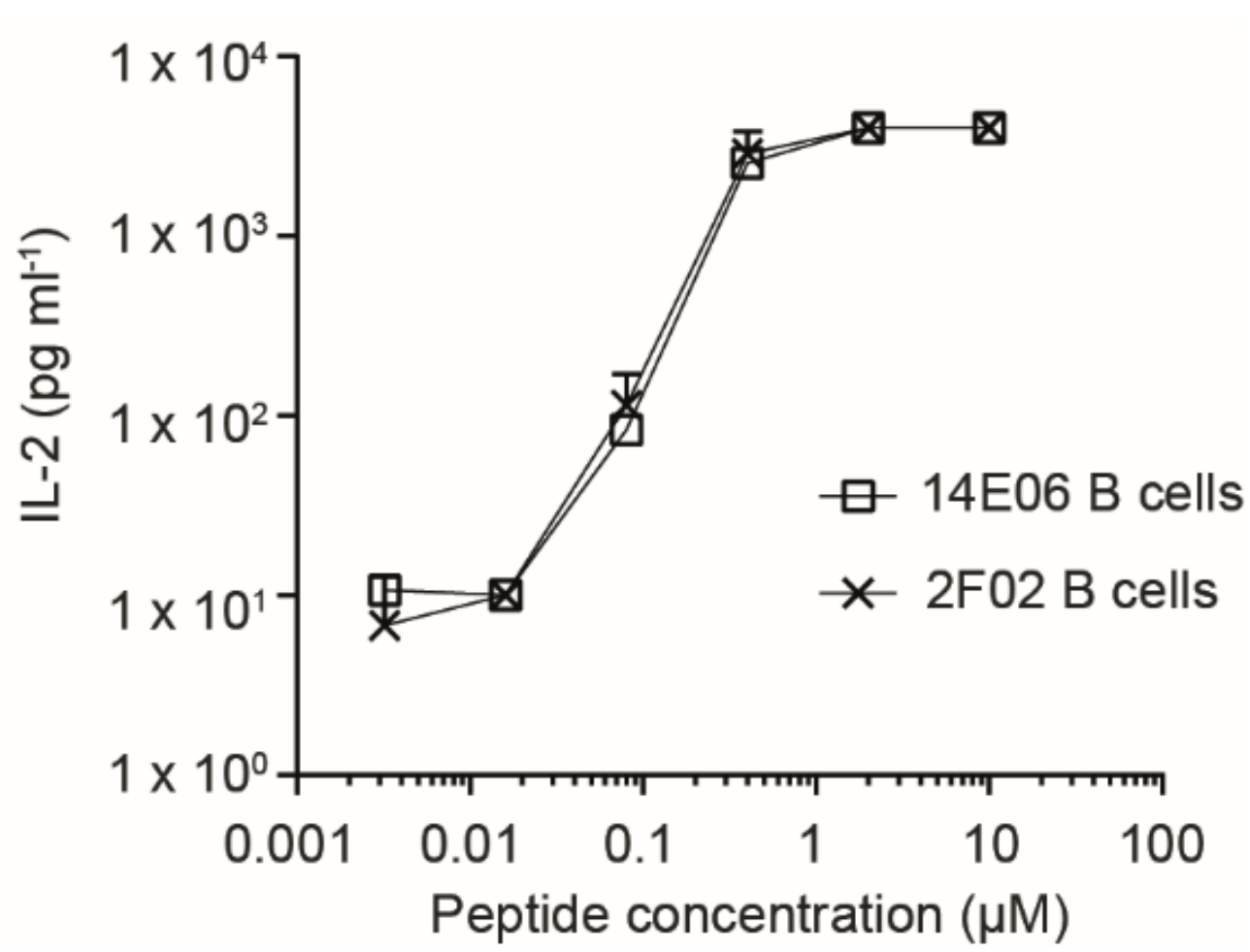

Supplementary Figure 2. Proliferation of gluten-specific BW58 T cells to deamidated gluten peptide. BW58 T cells transduced to express the DQ2.5-glia-ω2-reactive TCR showed equal proliferation as measured by release of IL-2 when cocultured with HLA-DQ2.5-expressing TG2-specific (14E06) or non-specific (2F02) A20 B cells in the presence of deamidated ω18 gliadin peptide (QPEQPFPQPEQPFPWQPQ). Symbols represent mean IL-2 concentration with indication of standard deviation based on culture triplicates.

Supplementary Figure 3:

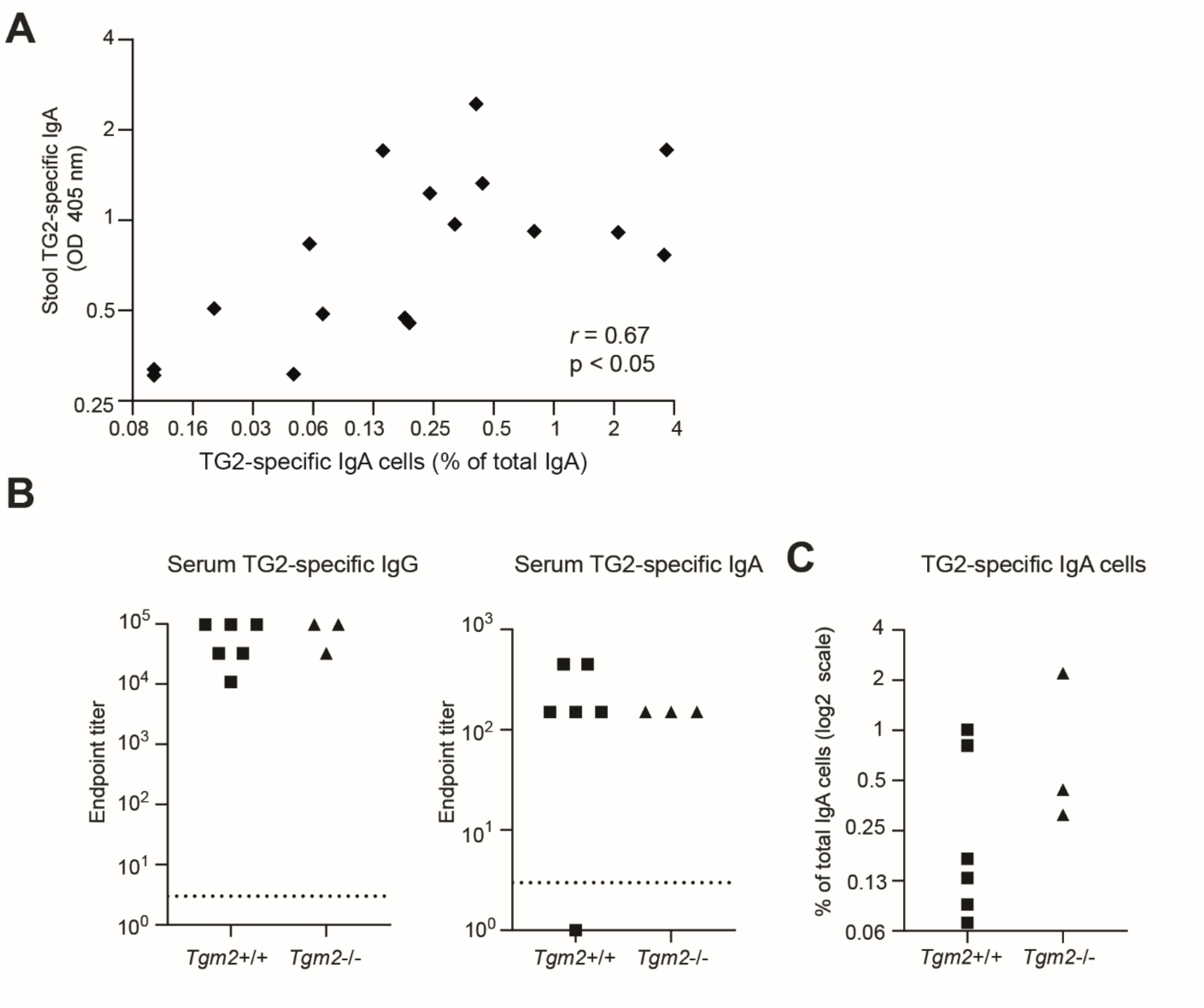

Supplementary Figure 3. Anti-TG2 IgA responses in *Tgm2*+/+ and *Tgm2*-/- HLA-DQ2.5 KI recipient mice immunized with α-14E06-ω18 Troy and CT. (*A*) Correlation between the frequency of TG2-specific IgA plasma cells in the small intestinal lamina propria and anti-TG2 IgA levels in stool samples on day 20 in *Tgm2*+/+ HLA-DQ2.5 KI immunized as described in Figure 2*A*. *r*: Spearman's correlation coefficient. (*B*) HLA-DQ2.5 KI mice on *Tgm2*+/+ or *Tgm2*-/- background were adoptively transferred with TG2-specific B cells isolated from *Tgm2*-/- HLA-DQ2.5$^+$ 14E06 KI mice along with gluten-specific CD4$^+$ T cells isolated from TCR-glia-ω2 transgenic mice and subsequently immunized with mIgG2b α-14E06-ω18 Troy and CT for three rounds with weekly intervals. Serum was collected on day 19 after the first immunization, and anti-TG2 IgG and IgA levels were determined by ELISA. ELISA endpoint titer was defined as the reciprocal of the last dilution giving an absorbance above the cut-off. (*C*) Quantification of anti-TG2 IgA plasma cells per total IgA plasma cells in the small intestinal lamina propria as determined by ELISPOT. Data are pooled from two independent experiments.

Supplementary Figure 4:

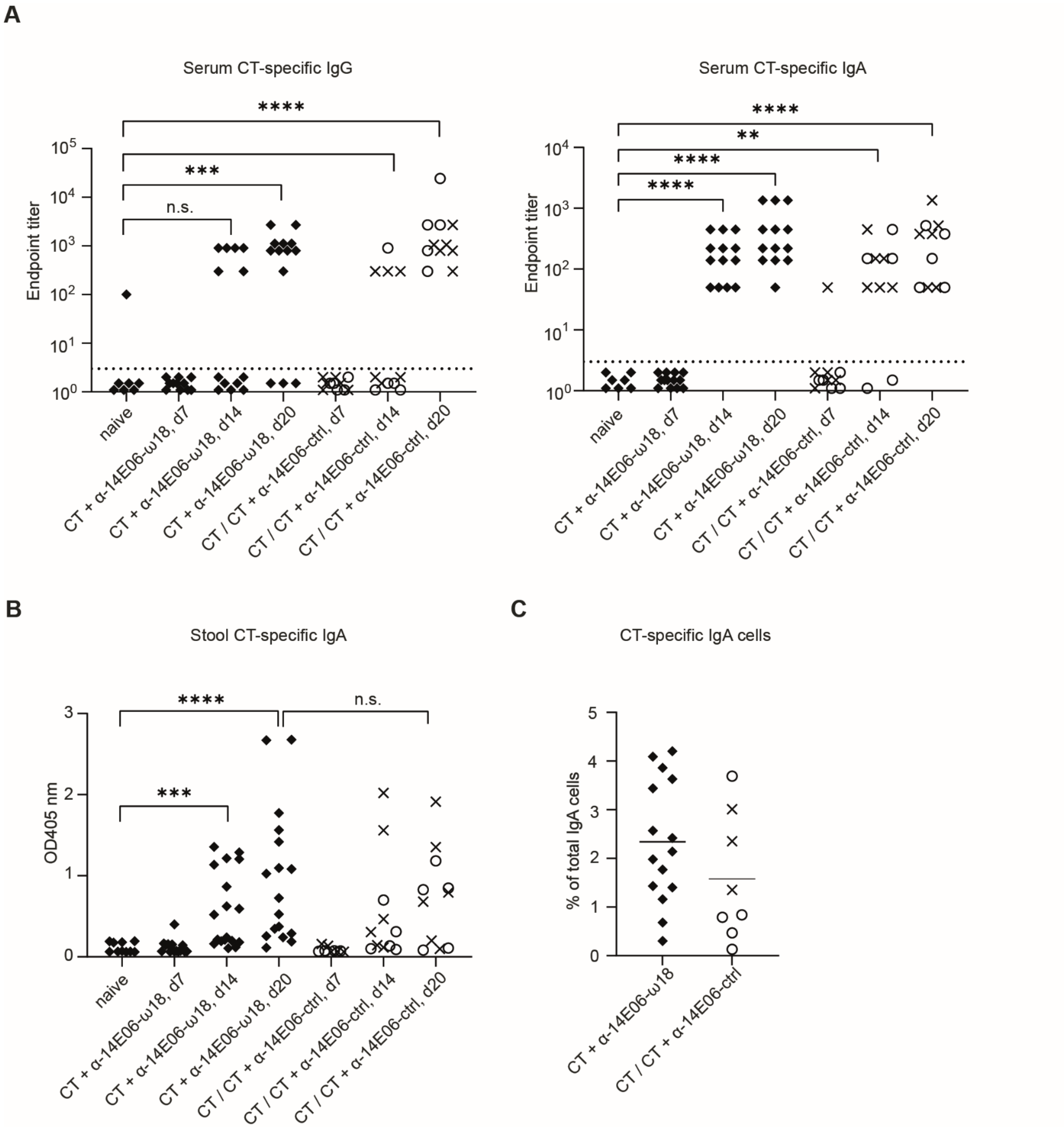

Supplementary Figure 4. Anti-CT responses upon oral immunization with mIgG1 α-14E06-ω18 Troy and CT. (*A*) ELISA endpoint titer quantification of CT-specific serum IgG and IgA antibodies in HLA-DQ2.5 KI mice with adoptively transferred TG2-specific B cells and gluten-specific $CD4^+$ T cells immunized i.g. with α-14E06-ω18 Troy and CT (diamonds), α-14E06-ctrl and CT (crosses), or CT alone (circles). ELISA endpoint titer was defined as the reciprocal of the last dilution giving an absorbance above the cut-off. (*B*) CT-specific IgA antibodies in stool (diluted 1:2) of mice orally immunized with α-14E06-ω18 Troy and CT (diamonds), α-14E06-ctrl and CT (crosses), or CT alone (circles). (*C*) ELISPOT quantification of CT-specific IgA plasma cells per total IgA plasma cells in the small intestinal lamina propria from mice immunized with mIgG1 anti-14E06-ω18 troybody and CT, mIgG1 α-14E06-ctrl and CT or CT alone. Data are pooled from 4 independent experiments. Error bars indicate SD. Median of each group was compared between groups with Mann-Whitney U test. (*), (**), (***), (****) indicate significant differences with $P<0.05$, $P<0.005$, $P<0.0005$ and $P<0.0001$, respectively. n.s.; not significant.

Supplementary Figure 5:

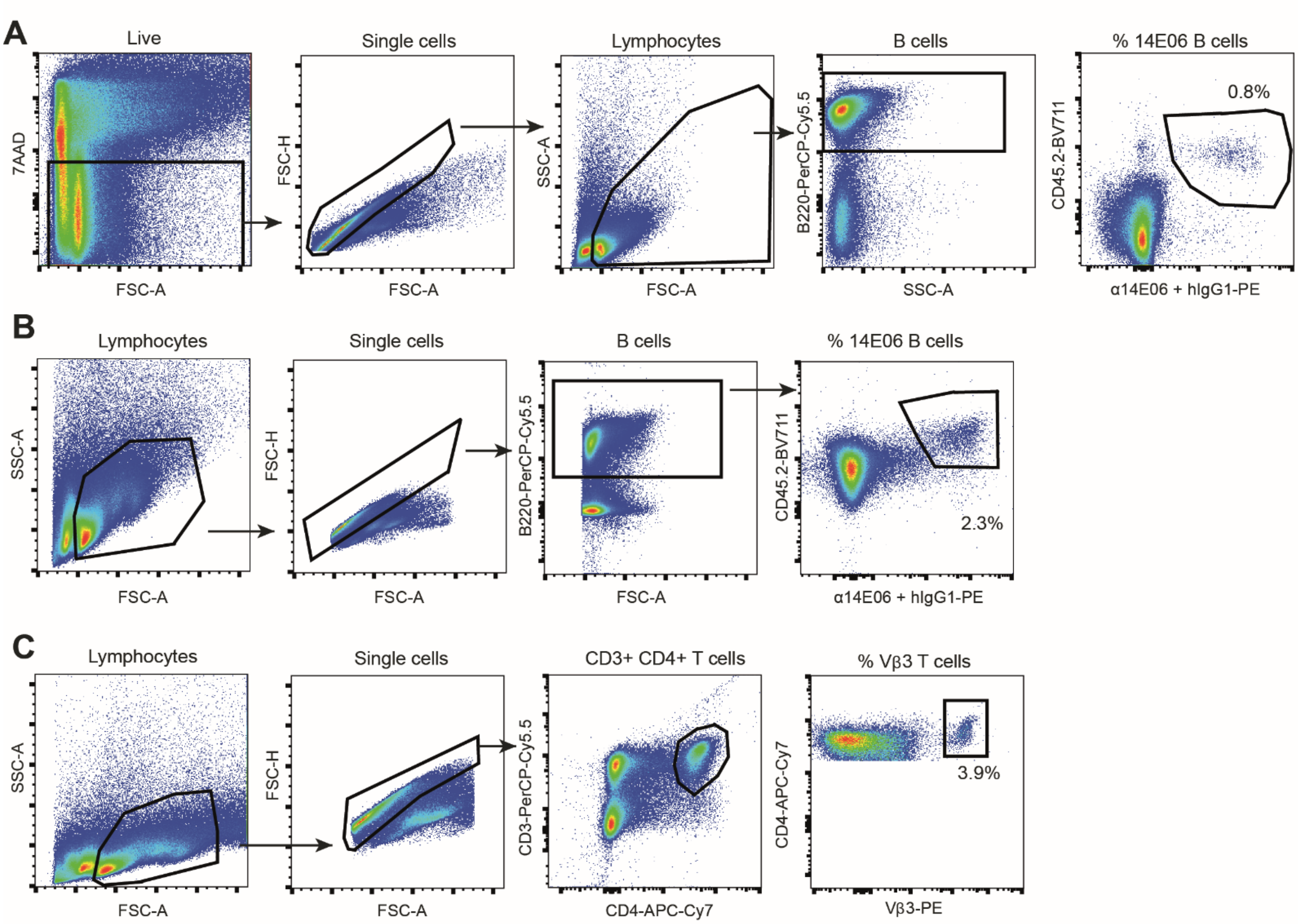

Supplementary Figure 5. Gating strategy of adoptively transferred T and B cells in PP and MLN. (*A*) Flow cytometry plots showing staining of single cell suspensions of Peyer's patches of a CD45.1/1 HLA-DQ2.5 KI mouse adoptively transferred with CD45.2/2 TG2-specific B cells and gluten-specific CD4^{+} T cells as shown in Fig. 2a. The analysis shows day 20 after three rounds of immunization with mIgG1 α-14E06-ω18 Troy and CT. (*B*) Flow cytometry plots of MLN on day 20 showing the gating of adoptively transferred TG2-specific (α-14E06^{+}) B cells. (*C*) Flow cytometry plots of MLN on day 14 after two rounds of immunization with mIgG1 α-14E06-ω18 Troy and CT showing the gating of transferred gluten-specific T cells expressing the transgenic human Vβ3^{+} TCR.
